# An empirical investigation of taxi driver response behavior to ride-hailing requests: A spatio-temporal perspective

**DOI:** 10.1371/journal.pone.0198605

**Published:** 2018-06-08

**Authors:** Ke Xu, Luping Sun, Jingchen Liu, Hansheng Wang

**Affiliations:** 1 Guanghua School of Management, Peking University, Beijing, P.R.China; 2 Business School, Central University of Finance and Economics, Beijing, P.R.China; Universidad de Castilla-La Mancha, SPAIN

## Abstract

Using data provided by a ride-hailing platform, this paper examines the factors that affect taxi driver response behavior to ride-hailing requests. The empirical investigation from a driver’s perspective is of great importance for ride-hailing service providers, given that approximately 40% of the hailing requests receive no response from any driver. To comprehensively understand taxi driver response behavior, we use a rich dataset to generate variables related to the spatio-temporal supply-demand intensities, the economic incentives, the requests’ and the drivers’ characteristics. The results show that drivers are more likely to respond to requests with economic incentives (especially a firm subsidy), and those with a lower spatio-temporal demand intensity or a higher spatio-temporal supply intensity. In addition, drivers are more likely to respond to requests involving rides covering a greater geographical distance and to those with a smaller number of repeated submissions. The drivers’ characteristics, namely, the number of requests received and the number of requests responded, however, have relatively little impacts on their response probability to the current request. Our findings contribute to the related literature and provide managerial implications for ride-hailing service providers.

## Introduction

The rapid development of the Internet and mobile communication technology has promoted the integration of online technology and offline businesses, creating a brand-new business model, i.e., the Online to Offline (O2O) business [1]. In O2O businesses, consumers search and pay for services or products online, but receive and consume them offline. This business model takes advantage of the Internet as a marketing channel and helps firms seize more offline business opportunities. In recent years, O2O has become another growing trend after Business to Consumer (B2C) and Consumer to Consumer (C2C) e-commerce [2], and has been widely adopted in services such as beauty, catering, housekeeping, and movies, among others. Well-known brands based on O2O include Airbnb, Deliveroo, Postmates, Uber, etc. According to *iResearch Consulting Group*, the revenue of O2O businesses in China is expected to reach 978 billion RMB in 2017, and its annual growth rate has been above 40% for several years [3].

We use an example to illustrate the difference between traditional businesses and O2O businesses. In China (especially in urban areas), traditional businesses often use sticker-type small advertisements to reach and acquire customers. The advertised products or services include but are not limited to, catering, renting and housekeeping. These kinds of advertisements are inefficient and provide consumers with little information about quality. With the help of the Internet, however, O2O mobile applications (apps) such as 58Daojia (www.daojia.com) are able to effectively organize and integrate huge amounts of supply information on a single e-platform. This provides customers with more convenient access to select a desired supplier. In addition, some O2O apps can identify the service suppliers that each individual customer is likely to be interested in and send related information to the customer. Through this approach, customers can be better served and service quality can be greatly improved.

O2O businesses are essentially two-sided markets that match the C-side (Consumer) demand with the B-side (Business) supply. They provide an e-platform (e.g., a mobile app) to facilitate access for C-side and B-side. Accurately matching supply with demand from both sides is the key to success for O2O businesses, because they profit by charging a commission on the transactions made on their platforms. Thus, the more accurate the matching they offer, the more revenue the platform obtains.

In this study, we focus on one of the most representative services that benefit from O2O: ride-hailing service. Traditionally, passengers flag passing taxis on the street, while taxi drivers drive around looking for potential passengers. However, this traditional way of ride-hailing has many inefficiencies. For passengers, it is often difficult to hail a taxi in rush hours or in locations with high passenger demands. In contrast, for taxi drivers, the traditional ride-hailing approach can lead to low loading efficiency, and a significant portion of their time can be spent driving a vacant taxi.

The emergence of ride-hailing apps has tremendously improved ride-hailing efficiency. A ride-hailing app is a smartphone-based e-platform capable of geographically locating both taxi drivers and passengers, and then efficiently matching drivers with passengers. The well-known ride-hailing apps include Lyft (www.lyft.com) and Uber (www.uber.com) in the US, and Didi Chuxing (www.didichuxing.com) and Yidao (www.yongche.com) in China. Using Didi Chuxing as an example. Passenger who needs a ride can simply open the Didi Chuxing app, type in a specific departure and destination, and finally submit the hailing request by clicking the button “Call for a Taxi”. The app automatically locates the passengers and taxi drivers through its GPS system on a real-time basis. It then sends the hailing request to nearby taxi drivers. Upon receiving the request, the taxi drivers decide whether to respond to the request. The ride-hailing app chooses one driver to get the deal. If no driver responds, the request fails and is aborted. Most ride-hailing platforms in China (e.g., Didi Chuxing) work in this manner. Note that Uber adopts a dispatch system in which drivers are automatically assigned to (rather than freely choose) hailing requests. This research focuses on taxi driver response behavior to ride-hailing requests; analysis of a dispatch system is beyond the scope of this research.

A well-designed ride-hailing app can match passengers and taxi drivers efficiently using some algorithms, significantly reducing search costs for both parties. The value that a ride-hailing app creates, as well as customer satisfaction with the app, largely depends on whether the hailing request can be fulfilled by a taxi driver. Consequently, the driver response rate to hailing requests is a key performance indicator (KPI) of the ride-hailing service providers. Even for ride-hailing apps with a very large driver base (e.g., Didi Chuxing), the driver response rate is notoriously low; thus, many hailing requests go unfulfilled.

According to the 2015 Hail Market Analysis of New York City, in the first half of the year, 99,440 e-hail requests went unfulfilled outside the Manhattan core area [4]. Other evidence shows that 41% of the ride-hailing requests in New York went unfulfilled in 2014. Although this percentage shrank to 18% in 2015 [5], it is still not a small number. The unmet ride-hailing demand in China is even higher. On January 23rd of 2017, more than 40% of the ride-hailing requests on Didi Chuxing in Shanghai received no response from any driver [6] and this is not an exception. According to the data published by Didi Chuxing in 2017, nabbing a vehicle in big cities, such as Beijing, Shanghai, Guangzhou, and Shenzhen, has significantly increased in difficulty [7], even during the non-peak hours [8]. Specifically, the response rate for rides hailed at busy pickup spots such as airports, train stations, hospitals, and schools has declined noticeably. The response rates for rides hailed from similar spots in second- and third-tier cities have decreased by an average of 30% annually [7]. For ride-hailing service providers such as Didi Chuxing, these unfulfilled requests represent a huge loss, not merely in terms of the transaction value that could otherwise be achieved but also in terms of the damage to passenger loyalty [9]. In a two-sided market, the market base of the demand side is vital to the maintenance and expansion of the supply side (i.e., the drivers in our case) [10].

Therefore, understanding the factors that affect driver response behavior to hailing requests is a substantial problem for ride-hailing service providers. Given the importance of this research question, it is surprising that, to our knowledge, very few researchers have examined ride-hailing services from a driver’s perspective. Some earlier studies used analytical models to examine the consequences of the surge pricing policy or the competition among different ride-hailing platforms [11, 12]. Other studies have developed empirical models to more accurately predict the demand for rides [13–15]. Still others used the massive data collected by ride-hailing apps to predict the real-time ride-hailing supply-demand gap [16]. Few studies, however, have examined driver response behavior to ride-hailing requests.

In this study, we derived the real-time ride-hailing supply and demand intensities from a rich dataset and examined how the spatio-temporal dynamics systematically influence driver response behavior to ride-hailing requests. To the best of our knowledge, this is the first empirical work that examines this research question. In addition to the spatio-temporal dynamics, we examined whether economic incentives (i.e., firm subsidies and passenger premiums) are effective in increasing the driver response rate. Answering these questions would provide implications for the theoretical literature as well as for ride-hailing service providers.

## Data description

### Data sources

The data used in this study is provided by one of the well-known ride-hailing service providers in China. Our research complies with the terms of use of the ride hailing platform. The ride-hailing platform provided us all the request records in the urban area of Beijing on a working day in March, 2015. Beijing is not only the capital city but also the political, economic and cultural center of China, and the the world’s third most populous city as well. Beijing is governed as a direct-controlled municipality with 16 urban, suburban, and rural districts [17], and the transportation is very convenient with six ring roads covering the whole urban area of the city. Our data includes 248,757 ride-hailing requests submitted within the sixth ring road of Beijing. Note that the requests include both fulfilled and unfulfilled requests, depending on whether the drivers respond. To better understand how a driver response is generated, the details of the ride-hailing process are given below [18, 19].

**Step 1.** Passengers who need a ride log into the ride-hailing app. The app uses its GPS system to automatically identify the passenger’s current location and then the passenger types in the destination.
**Step 2.** The passenger determines whether to offer a premium to the driver. In most situations, the passengers do not offer any premiums. However, during rush hours or in high demand locations, it can be extremely difficult to get a ride without offering a decent premium. After determining the premium amount, the passenger submits the request to the app.
**Step 3.** After the app receives the request, it determines whether and how much to offer as a subsidy to the request. Then, it sends the request to a number of taxi drivers within a reasonable distance through its voice-based system. The voice-based system will broadcast the departure and destination locations of the request, whether the passenger offers a premium, how much subsidy the firm offers, etc.
**Step 4.** Upon receiving the request, the drivers decide whether to respond. The ride-hailing app chooses one driver to get the deal based mainly on how quick the driver responds, how far away the driver is, and how convenient it is for the driver to pick up the passenger. When no driver responds to a request, it will be cancelled.

Consistent with the numbers reported by the industry, our data showed that only 60.1% of the ride-hailing requests received responses; the remaining 39.9% did not. This finding suggests that, even with the “match-making” service of the ride-hailing app, a significant portion of the requests were not fulfilled by any drivers. Thus, it is relevant and important to help practitioners improve the driver response rate. We next examine how the driver response rate differs across time and locations, and present evidence for the importance of modeling the spatio-temporal dynamics.

### Spatio-temporal dynamics of supply-demand imbalance

**Fig 1:**
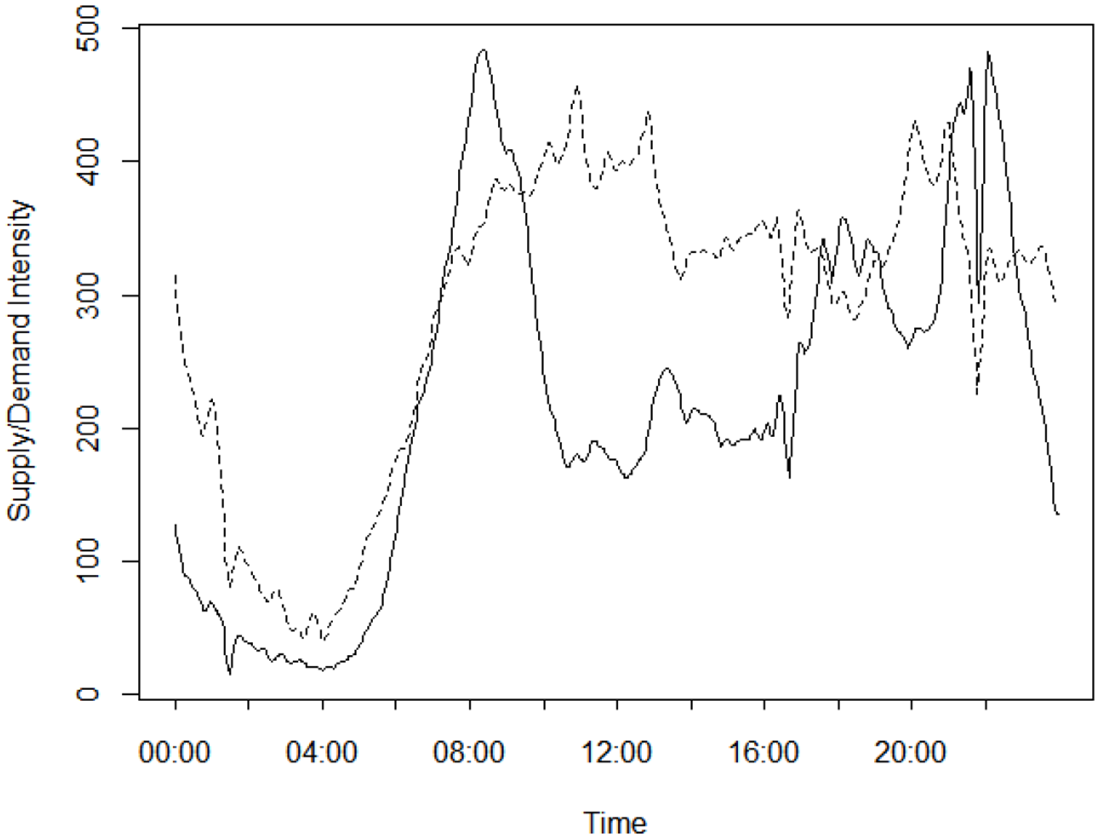
Supply and demand intensities over time. The solid line indicates the demand intensity (measured by the total number of hailing requests), and the dashed line represents the supply intensity (measured by the number of available drivers).

In this subsection, we apply some descriptive statistics to illustrate the gap between ride-hailing supply and demand across time and locations. This may help to explain low driver response rates to ride-hailing requests. We first examine the supply-demand imbalance over time. To this end, we plot the supply and demand intensities during different times of the day in Fig 1. This figure suggests that the supply-demand imbalance differs drastically over different time periods. In particular, the demand can far exceed the supply (i.e., the dashed line is below the solid line) in the early peak hours (i.e., 06:00–10:00), the late peak hours (i.e., 17:30–19:30), and the evenings (i.e., 21:00–22:30). During these periods, many requests may not receive responses from any drivers; thus, the passengers cannot get a ride. In contrast, supply exceeds the demand in the early morning (i.e., before 06:00) and from 10:00 to 17:30. During these periods, requests may receive responses from more than one driver. As a result, many taxis may remain vacant.

**Fig 2:**
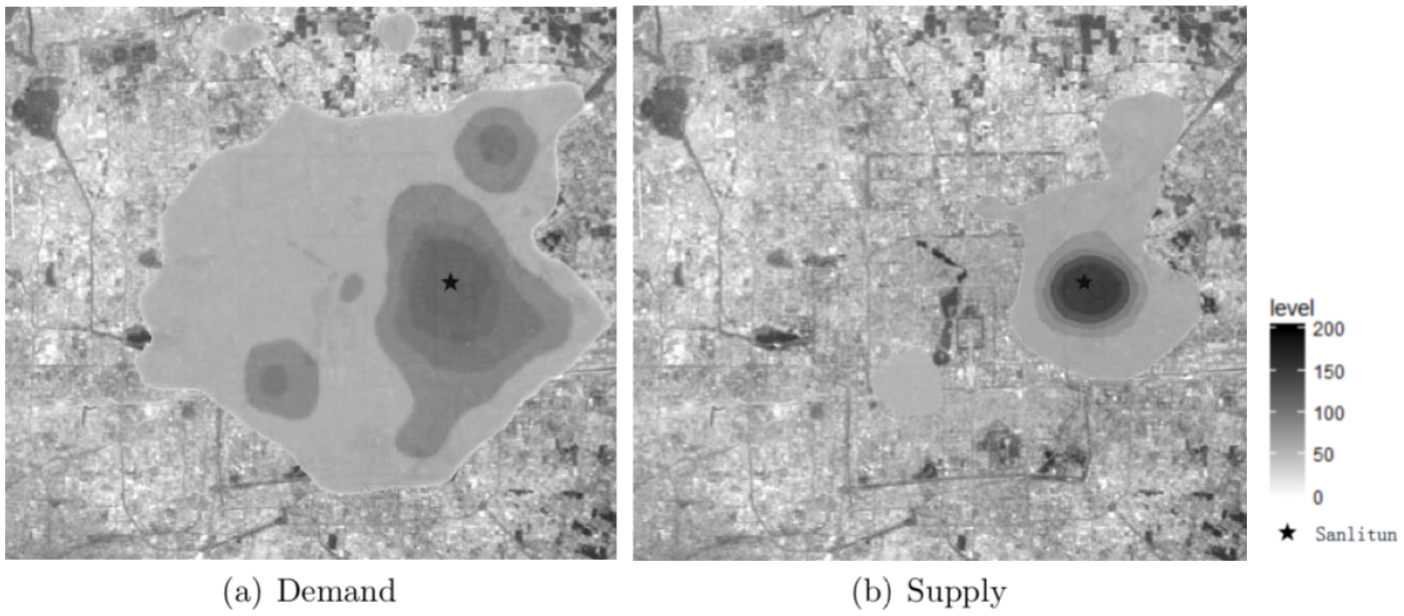
Thermodynamic analysis of spatial (a) demand and (b) supply. The darker areas are locations with higher demand/supply intensities. The distributions of demand and supply are quite different across the city. For example, in the area around Sanlitun, the supply intensity is the highest, while the demand intensity is relatively low. This supply-demand imbalance may lead to a low response rate from taxi drivers.

We further examine the supply-demand imbalances in different locations. During different time periods, the supply-demand intensities differ substantially from one location to another. For example, we plot the demand and supply intensities from 00:00 to 06:00 in Fig 2, which shows the urban area of Beijing, ranging from Lat 40°01’19”N, Lon 116°15’16”E to Lat 39°50’42”N, Lon 116°30’01”E. The original image is No. STS090-714-42 from NASA (Image courtesy of the Earth Science and Remote Sensing Unit, NASA Johnson Space Center. URL: http://eol.jsc.nasa.gov). As we can see, Beijing spreads out in six concentric ring roads, and the main urban area of this city is within the fifth ring road [17]. The demand is dispersed across the urban area of the city (see Fig 2(a)), while the supply is highly concentrated in the area of Sanlitun, which lies in the east third ring road and is a popular commercial area for shopping, dining and entertainment (see Fig 2(b)). Therefore, it is easier for passengers around Sanlitun to hail a taxi (i.e., get a response from the taxi drivers), while passengers in other areas may find it very difficult to get a response from a taxi driver.

This subsection provides some model-free evidence regarding the relationship between the temporal and spatial supply-demand imbalances and driver response rate to ride-hailing requests. This motivates us to examine driver response behavior from a spatio-temporal perspective. To this end, the spatio-temporal related explanatory variables are described in the next subsection.

### Variable description

We derive five sets of variables that may influence driver response behavior (see Table 1 for a summary). These variables capture the spatio-temporal dynamics of supply and demand intensities, the economic incentives offered to the drivers, the characteristics of the requests, the characteristics of the drivers, and the time effects, respectively.

**Table 1:** Description of explanatory variables. There are five sets of explanatory variables, corresponding to spatio-temporal supply-demand intensities, economic incentives, request characteristics, driver characteristics, and the time factor.

Variable Attribute	Variable Name	Notation	Value	Baseline
Spatio-temporal Supply-demand Intensities	Demand Intensity	*X*_1_	Continuous variable	-
	Supply Intensity	*X*_2_	Continuous variable	-
Economic Incentives	Passenger Premium	*X*_3_	*X*_3,1_: 3, 5 RMB;	No premium
			*X*_3,2_: >5 RMB.	
	Firm Subsidy	*X*_4_	*X*_4,1_: 1–5 RMB;	No subsidy
			*X*_4,2_: >5 RMB.	
Characteristics of Hailing Requests	Geographical Distance	*X*_5_	Continuous variable	-
	Number of Repeated Submissions	*X*_6_	Continuous variable	-
Characteristics of Drivers	Number of Requests Received	*X*_7_	Continuous variable	-
	Number of Requests Responded	*X*_8_	Continuous variable	-
Time Factor	Request Time	*X*_9_	*X*_9,1_: 00:00–02:00;	22:00–24:00
			*X*_9,2_: 02:00–04:00;	
			*X*_9,3_: 04:00–06:00;	
			*X*_9,4_: 06:00–08:00;	
			*X*_9,5_: 08:00–10:00;	
			*X*_9,6_: 10:00–12:00;	
			*X*_9,7_: 12:00–14:00;	
			*X*_9,8_: 14:00–16:00;	
			*X*_9,9_: 16:00–18:00;	
			*X*_9,10_: 18:00–20:00;	
			*X*_9,11_: 20:00–22:00.	

First, spatio-temporal dynamics are captured by the real-time supply and demand intensities nearby when each hailing request is submitted. For each request, the spatio-temporal demand density *X*_1_ is operationalized as the total number of concurrent requests within one square kilometer during the five minutes before the request. Similarly, the spatio-temporal supply density *X*_2_ is captured by the total number of drivers within one square kilometer during the five minutes before the request.

Regarding economic incentives, the dataset provides us with two variables: passenger premium *X*_3_ and firm subsidy *X*_4_. Passenger premium refers to the tip that the passenger is willing to pay to the driver, which may take values of 0, 3, 5, 10 or 20 RMB. We recode this variable into two dummy variables: *X*_3,1_ represents a premium of 3, 5 RMB and *X*_3,2_ corresponds to a premium more than 5 RMB. “No premium” serves as the baseline. In addition to the premium offered by passengers, the ride-hailing firm may also offer some subsidy to the drivers for each order completed. The subsidy may be 0, 1, 2, 3, 4, 5, 6 RMB or more, and the amount is determined by the firm. We recode this variable into two dummy variables: *X*_4,1_ indicates a subsidy of 1–5 RMB and *X*_4,2_ represents a subsidy of more than 5 RMB. “No subsidy” serves as the baseline.

Whether taxi drivers respond to a hailing request may also depend on the characteristics of the request. The dataset includes a continuous variable *X*_5_ that denotes the geographical distance between the departure and the destination locations. The attractiveness of an order may depend on the geographical distance of the request. The other variable *X*_6_ is the number of times that a request has been repeatedly submitted by the same passenger.

We generate two variables to capture the status of the drivers at the time of receiving a particular hailing request. The first is the total number of requests received by the driver in the five minutes before the current request. This variable captures the number of alternative requests that the driver may have considered responding to, denoted as *X*_7_. The more alternative requests a driver receives, the greater the interference to the driver, making it less likely that the driver will respond to the current request. Second, we also compute the number of requests responded to by the driver in the five minutes before the current request. This variable indicates how active the driver is in responding to hailing requests immediately before receiving the current request and is denoted as *X*_8_. Because more active drivers may be more likely to respond to subsequent requests, we conjecture that this variable may significantly influence the driver’s response behavior toward the current request.

Finally, to control for time effects, we divide the 24-hour day into 12 time slots and generate 11 dummy variables accordingly. Each time slot represents a two-hour period of the day. Starting from 00:00–02:00, the time slots are labeled consecutively, ranging from *X*_9,1_ to *X*_9,11_. The time slot 22:00–24:00 serves as the baseline. Including these dummy variables in the model allows us to accommodate possible nonlinear effects.

## Statistical analysis

### Descriptive analysis

The descriptive statistics of the explanatory variables are shown in Table 2. On average, the spatio-temporal demand intensity (7.4) is lower than the spatio-temporal supply intensity (12.2), but their standard deviations are large, indicating that the spatio-temporal supply and demand intensities vary considerably across different requests. Regarding the variables related to economic incentives, most hailing requests in our data carry no passenger premium or firm subsidy. In addition, the requests are almost equally distributed among different time slots: peak-hour slots have slightly more requests, whereas there are relatively few requests during the night time slots.

**Table 2:** Summary statistics of explanatory variables. For each continuous variable, we show its mean, standard deviation, median, minimum, and maximum value, while for each categorical variable, we show the percentage of requests for each level of the variable. N = 15,339,333.

Variable	Value	Mean	SD	Median	Min	Max
*X*_1_	Continuous variable	7.4	9.0	4.0	1.0	135.0
*X*_2_	Continuous variable	12.2	17.3	7.0	0.0	391.0
*X*_3_	*X*_3,1_: 3, 5 RMB;			9.3%		
	*X*_3,2_: >5 RMB;			0.1%		
	Baseline: No premium.			90.6%		
*X*_4_	*X*_4,1_: 1–5 RMB;			18.4%		
	*X*_4,2_: >5 RMB;			0.9%		
	Baseline: No subsidy.			80.7%		
*X*_5_	Continuous variable	6.8	5.4	5.3	1.0	32.8
*X*_6_	Continuous variable	1.1	0.4	1.0	1.0	12.0
*X*_7_	Continuous variable	11.7	9.9	10.0	1.0	89.0
*X*_8_	Continuous variable	5.8	6.2	4.0	0.0	39.0
*X*_9_	*X*_9,1_: 00:00–02:00;			3.4%		
	*X*_9,2_: 02:00–04:00;			1.3%		
	*X*_9,3_: 04:00–06:00;			1.0%		
	*X*_9,4_: 06:00–08:00;			5.9%		
	*X*_9,5_: 08:00–10:00;			10.0%		
	*X*_9,6_: 10:00–12:00;			10.3%		
	*X*_9,7_: 12:00–14:00;			10.7%		
	*X*_9,8_: 14:00–16:00;			10.4%		
	*X*_9,9_: 16:00–18:00;			11.7%		
	*X*_9,10_: 18:00–20:00;			12.8%		
	*X*_9,11_: 20:00–22:00;			12.8%		
	Baseline: 22:00–24:00.			9.8%		

**Fig 3:**
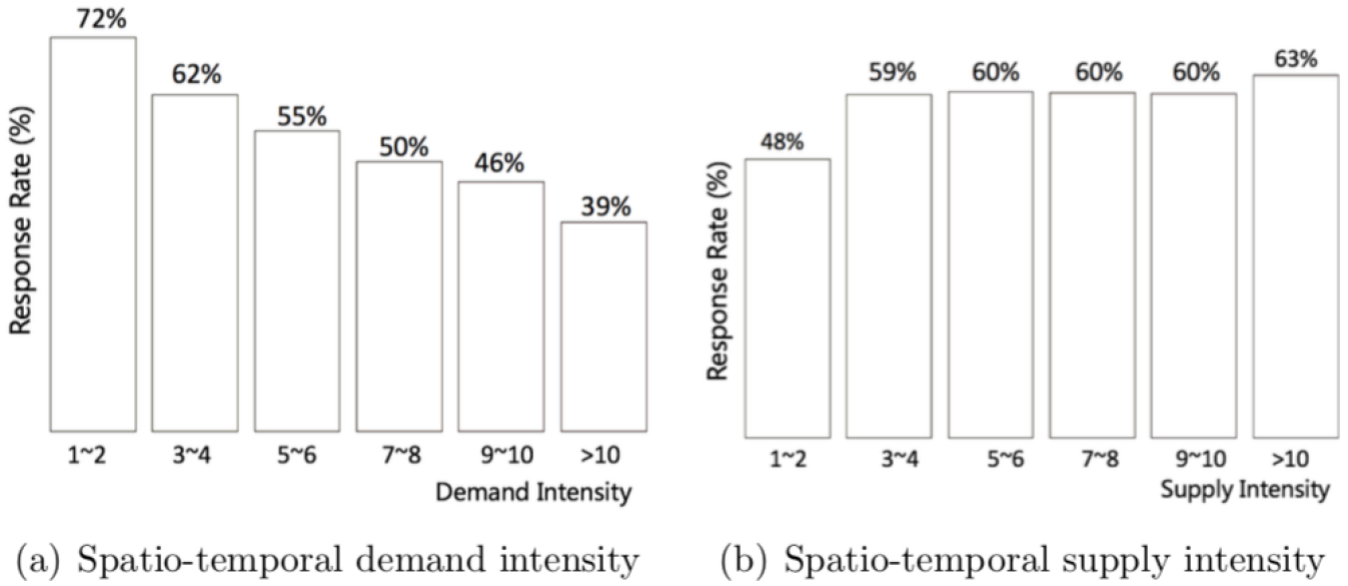
Response rate and spatio-temporal demand and supply intensities. (a) The driver response rate declines steadily as the spatio-temporal demand intensity increases, (b) while it initially increases but then becomes stable as the spatio-temporal supply intensity increases.

We further explore the potential relationship between each set of explanatory variables and taxi driver response rate. For the spatio-temporal dynamics, we plot the driver response rate at different supply and demand intensities in Fig 3. We find that the spatio-temporal supply and demand intensities have opposite impacts on the driver response rate. In particular, the response rate declines steadily as the spatio-temporal demand intensity increases (see Fig 3(a)), but it increases as the spatio-temporal supply intensity grows. In addition, the results show that the response rate increases quickly when the supply intensity increases from “1–2” to “3–4”, but it becomes stable as the supply intensity increases further (see Fig 3(b)). Thus, the impacts of demand and supply intensities on response rate seem to be asymmetric.

**Fig 4:**
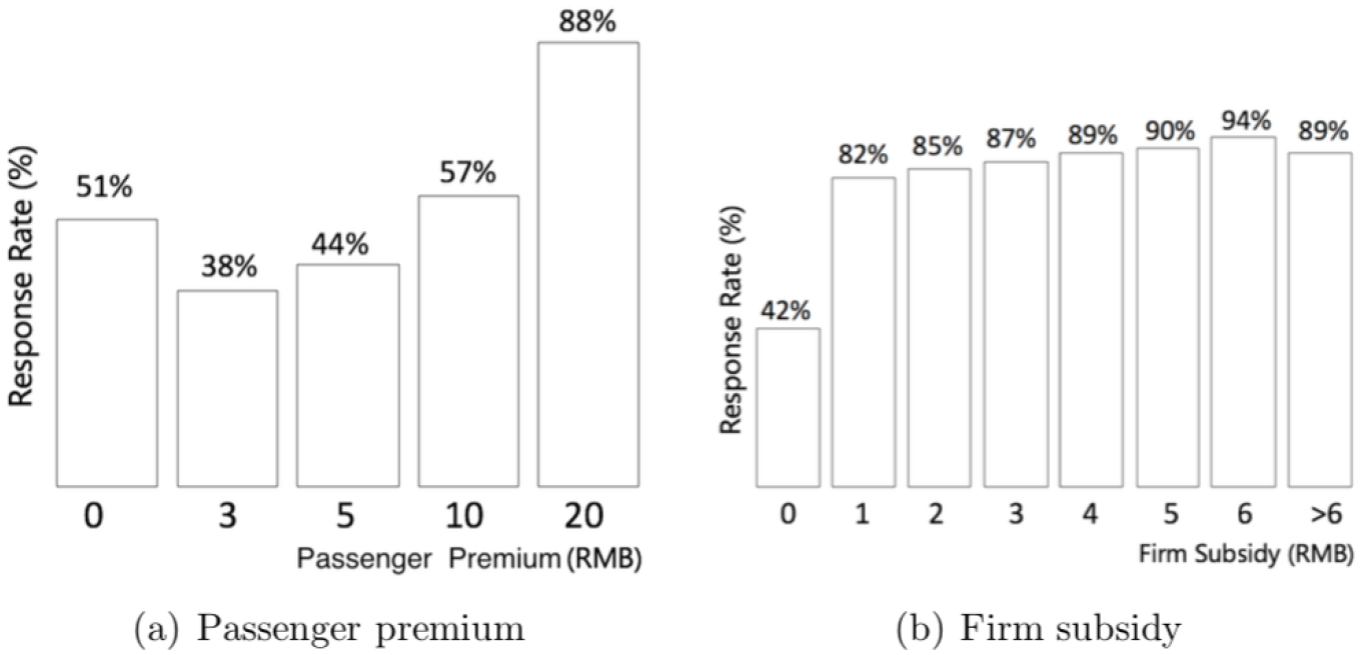
Response rate and economic incentives. (a) Passenger premium seems to have a “U-shaped” relationship with the driver response rate. (b) The presence (vs. absence) of a firm subsidy has a large positive impact on the driver response rate, which stabilizes when the amount of subsidy further increases.

The driver response rate at different levels of passenger premium and firm subsidy (i.e., economic incentives) is shown in Fig 4. Passenger premium seems to have a “U-shaped” relationship with the driver response rate. Fig 4(a) shows that either no premium or a very generous premium would lead to a relatively high response rate (e.g., up to 88% for requests with a premium of 20 RMB or more). Regarding firm subsidy, the presence (vs. absence) of a subsidy seems to matter considerably; even a 1 RMB subsidy increases the response rate from 42% to 82% (see Fig 4(b)). As the subsidy amount increases further, the driver response rate stabilizes (i.e., it does not increase accordingly). When a request is submitted to the ride-hailing app, its voice-based system will announce whether the request is an appointment or not, the departure and destination locations, as well as “*the firm offers a subsidy of X RMB*” if there is any subsidy. Since many requests have no subsidy, some drivers may respond to the request before they hear the specific amount of the subsidy. This justifies why even a 1 RMB subsidy is highly effective in increasing driver response rate.

**Fig 5:**
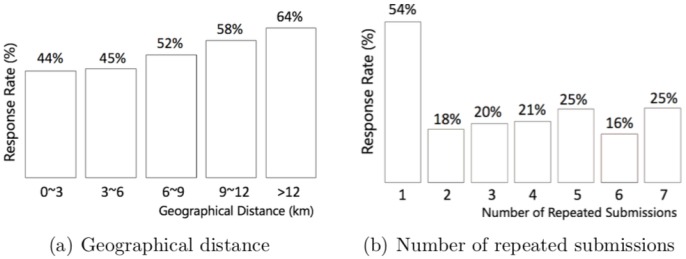
Response rate and request characteristics. (a) The driver response rate increases steadily as the geographical distance rises. (b) Regarding the number of repeated submissions, the response rate is the highest when a request is submitted only once to the ride-hailing platform, whereas it is comparatively low for repeatedly submitted requests.

The relationship between the characteristics of the request (i.e., geographical distance and the number of repeated submissions) and the driver response rate is plotted in Fig 5. Apparently, the driver response rate is higher for requests with longer geographical distances, indicating that these requests are more attractive to the drivers (see Fig 5(a)). This is perhaps because a longer geographical distance is often associated with a higher fare. Regarding the number of repeated request submissions, the driver response rate is the highest when the request is submitted only once to the ride-hailing platform (see Fig 5(b)). For requests repeatedly submitted by the same passenger, the driver response rate is comparatively low (ranging from 16% to 25%).

**Fig 6:**
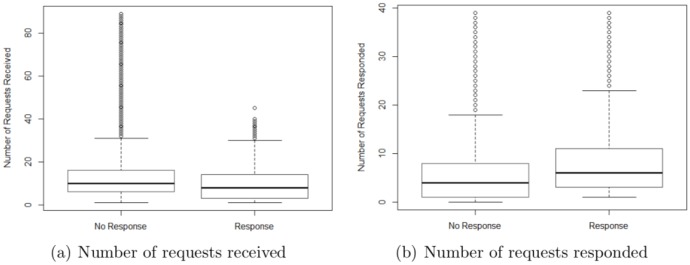
Response rate and driver characteristics. We denote the drivers who respond or do not respond to the current request as “Response” and “No Response” drivers, respectively. (a) The “Response” drivers tend to have received a smaller number of requests in the five minutes before the current request, (b) but they respond to a larger number of requests than the “No Response” drivers.

Regarding the driver characteristics, we plot the number of requests received and the number of requests responded in the five minutes before the current request for those drivers responding (i.e., Response) vs. not responding (i.e., No Response) to the current request (see Fig 6). As shown in Fig 6(a), the “Response” drivers tend to have received a smaller number of requests in the five minutes before the current request than the “No Response” drivers. Nonetheless, compared to the “No Response” drivers, the “Response” drivers responded to a larger number of requests in the five minutes before the current request (see Fig 6(b)). These results suggest that drivers who receive a smaller number of requests and who respond to a larger number of requests are more likely to respond to the current request.

**Fig 7:**
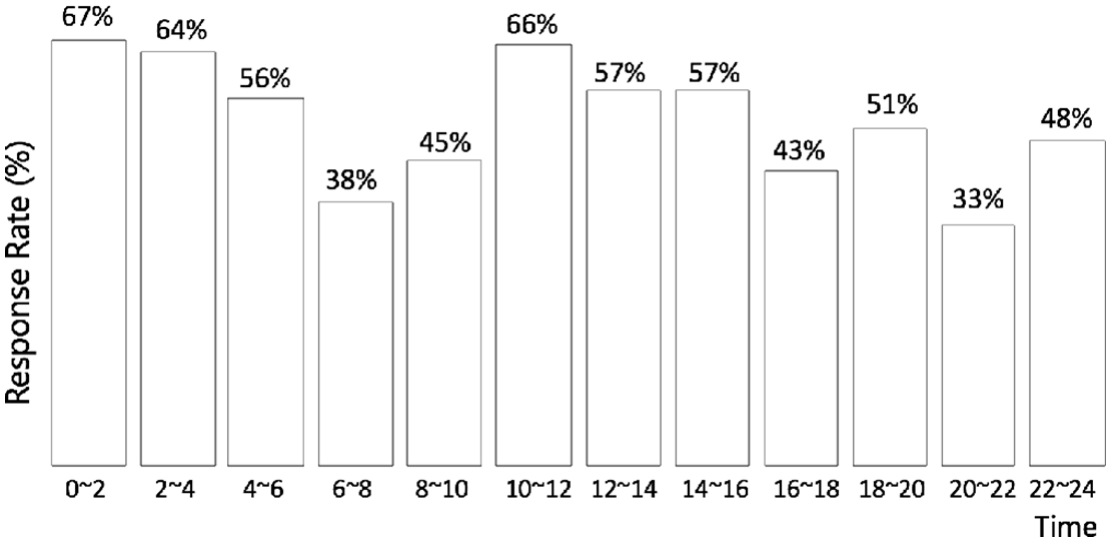
Response rate over time. The driver response rate is relatively low in the early peak hours (i.e., 06:00–10:00), the late peak hours (i.e., 16:00–18:00), and the evenings (i.e., 20:00–22:00), but considerably higher during the midnight and non-peak hours (e.g., 10:00–16:00).

Finally, Fig 7 shows the driver response rate over the 12 time slots of the day. Obviously, the response rate is relatively low in the early peak hours (i.e., 06:00–10:00), the late peak hours (i.e., 16:00–18:00), and the evenings (i.e., 20:00–22:00). During these periods, the ride-hailing demand (and the imbalance between supply and demand) is extraordinarily high; therefore, many requests are ignored by the drivers. In contrast, the response rate is relatively high during the midnight and non-peak hours (e.g., 10:00–16:00), possibly because the demand is relatively low. These observations are consistent with our previous analysis regarding the spatio-temporal dynamics of supply-demand imbalance in Fig 1.

### Modeling and results

Because a driver’s response to a particular request is a binary variable, we employ the logistic regression model to examine the effects of the explanatory variables. The model is specified as follows:

$$\begin{array}{cc} \text{Logit}\{P\left(Y_{ik}=1\right)\} & =\ln\left\{\frac{P(Y_{ik}=1)}{1-P(Y_{ik}=1)}\right\}\\ & =\beta_{0}+\beta_{1}X_{ik1}+\cdots +\beta_{p}X_{ikp}+\cdots +\beta_{P}X_{ikP},\\ & \;\;\;i=1,2,...\;,M,\;k=1,2,...\;,K(i). \end{array}$$ (1)

In Eq (1), *Y*_*ik*_ equals 1 if driver *k* responded to request *i* and 0 otherwise. *X*_*ikp*_ represents an explanatory variable *X*_*p*_ for request *i* and driver *k* (please refer to Table 1 for the details of the variables), and *β*_*p*_ represents the corresponding parameter to be estimated. *M* represents the total number of requests and *K*(*i*) represents the number of drivers receiving request *i*. In our data, the total number of observations *N* is $\sum_{i=1}^{M}K\left(i\right)=15,339,333$.

We use the maximum likelihood estimation (MLE) method to estimate the parameters. Because the distributions of the continuous variables are skewed and some continuous variables can be 0 (e.g., *X*_2_ and *X*_8_), we added “1” to all the continuous variables before taking the logarithm [20]. We then performed standardization to make the coefficients of the different explanatory variables comparable. We summarize the estimated coefficients in Table 3 alongside the corresponding standard errors, *Z*-values, and *p*-values. Below, we explain the impact of each set of explanatory variables after controlling for the other variables.

**Table 3:** Estimated regression coefficients. The dependent variable *Y*_*ik*_ is a binary variable that indicates whether driver *k* responded to request *k*.

	Coefficient	SE	*Z*-value	*p*-value
Intercept	-4.993	0.011	-468.486	<0.001
*X*_1_	-0.303	0.004	-86.345	<0.001
*X*_2_	0.246	0.003	75.774	<0.001
*X*_3,1_	0.045	0.009	5.154	<0.001
*X*_3,2_	1.431	0.386	3.710	<0.001
*X*_4,1_	0.612	0.005	115.898	<0.001
*X*_4,2_	0.878	0.017	52.116	<0.001
*X*_5_	0.348	0.002	139.948	<0.001
*X*_6_	-0.370	0.006	-66.756	<0.001
*X*_7_	-0.029	0.000	-91.024	<0.001
*X*_8_	0.065	0.000	201.344	<0.001
*X*_9,1_	0.420	0.020	20.978	<0.001
*X*_9,2_	0.491	0.024	20.393	<0.001
*X*_9,3_	0.757	0.015	52.058	<0.001
*X*_9,4_	0.780	0.012	65.664	<0.001
*X*_9,5_	0.508	0.012	43.077	<0.001
*X*_9,6_	0.088	0.012	7.107	<0.001
*X*_9,7_	0.198	0.012	16.706	<0.001
*X*_9,8_	-0.033	0.012	-2.719	0.007
*X*_9,9_	0.176	0.011	15.505	<0.001
*X*_9,10_	0.104	0.011	9.302	<0.001
*X*_9,11_	0.167	0.011	14.673	<0.001

*p*-value of Likelihood Ratio Test 0.000

As expected, the spatio-temporal supply and demand intensities have relatively large impacts on driver response behavior. The probability that a driver will respond decreases as the spatio-temporal demand intensity increases (${\hat{\beta }}_{1}$ = -0.303, *p* < 0.001). This phenomenon may occur because the intense nearby demand makes the drivers more willing to wait and search for better requests. In contrast, an increase in the spatio-temporal supply intensity significantly raises the driver response probability (${\hat{\beta }}_{2}$ = 0.246, *p* < 0.001). As the supply intensity increases (i.e., the presence of more competitors), the drivers become less picky and more likely to respond to requests. In addition, the difference between the absolute values of ${\hat{\beta }}_{1}$ and ${\hat{\beta }}_{2}$ is also significant (*p* < 0.001), suggesting that the demand and supply intensities have asymmetric impacts on driver response probability. Excessive demand tends to result in a lower driver response rate than does a shortage of supply.

Regarding economic incentives, firm subsidy seems to have a larger impact. In particular, compared with no subsidy, a subsidy of 1–5 RMB significantly increases the probability that a driver will respond to the request (${\hat{\beta }}_{4,1}$ = 0.618, *p* < 0.001). Increasing the subsidy beyond 5 RMB further significantly raises the probability that a driver will respond (${\hat{\beta }}_{4,2}$ = 0.878), but the additional increase (i.e., the difference between ${\hat{\beta }}_{4,1}$ and ${\hat{\beta }}_{4,2}$) is relatively small. Compared to firm subsidies, the other type of economic incentive, passenger premium, has a much smaller impact on the driver response probability. Compared with no premium, a premium of 3 and 5 RMB significantly increases the driver response probability (${\hat{\beta }}_{3,1}$ = 0.045, *p* < 0.001). A larger premium (i.e., more than 5 RMB) also leads to a large increase in driver response probability (${\hat{\beta }}_{3,2}$ = 1.431, *p* < 0.001).

Regarding the request characteristics, geographical distance has a large positive impact on driver response probability (${\hat{\beta }}_{5}$ = 0.348, *p* < 0.001). This finding suggests that drivers generally prefer requests that require traveling longer geographical distances, which is consistent with our descriptive analyses. The number of repeated submissions, however, has a large negative impact on driver response probability (${\hat{\beta }}_{6}$ = -0.370, *p* < 0.001), because drivers may infer that requests repeatedly submitted to the ride-hailing platform tend to be less attractive. Therefore, they are less likely to respond to these requests.

In contrast, the magnitude of the impact of the drivers’ characteristics is relatively small. Consistent with our descriptive analyses, the number of requests received by a driver significantly decreases the probability that the driver will respond to the current request (${\hat{\beta }}_{7}$ = -0.029, *p* < 0.001). The larger the number of requests received by a driver, the more alternatives that the driver has; consequently, the less likely it becomes that the driver will respond to the current request. The number of requests a driver responds to has a significant positive impact on the driver’s response probability for the current request (${\hat{\beta }}_{8}$ = 0.065, *p* < 0.001). The more active a driver is in responding to requests, the more likely it is that the driver will respond to the current request.

Finally, as control variables, the time dummy variables are also significantly correlated with driver response behavior. Everything else being equal, the driver response probability seems to be the highest between 04:00 and 08:00, immediately followed by the midnight period (i.e., 00:00–04:00) and the early peak hours (i.e., 08:00–10:00). In other time periods, the probability that drivers will respond is relatively low, especially between 14:00 and 16:00.

**Fig 8:**
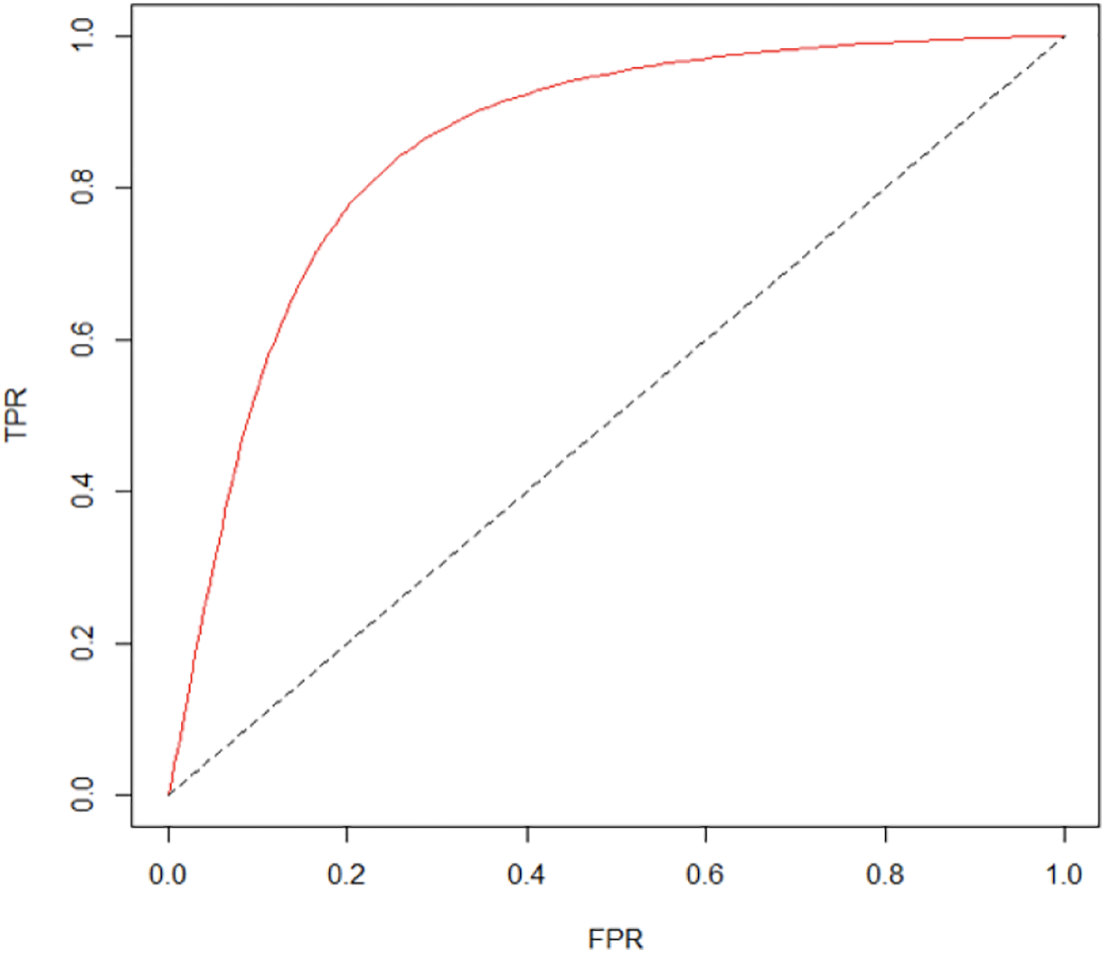
The ROC curve. FPR represents the false positive rate, and TPR is the true positive rate. The ROC curve is close to the upper left corner, indicating that the model’s predictive ability is good.

To assess the predictive power of our model, we adopt a useful tool for binary classifier evaluation—the Receiver Operating Characteristic (ROC) curve. The horizontal axis of the ROC curve represents the false positive rate (FPR), also known as “1—specificity”. In our research setting, FPR is the proportion of requests incorrectly predicted to be “Response” among all the “No Response” requests. The vertical axis is the true positive rate (TPR), also known as “sensitivity”. TPR refers to the proportion of requests correctly predicted to be “Response” among all the “Response” requests. Hence, a smaller FPR and a larger TPR indicate a higher predictive power. Fig 8 shows the ROC curve of our model, which is close to the upper left corner, implying that the predictive ability of the model is good.

To avoid overfitting, we randomly divided the data into a training set (80% of the observations) and a testing set (20% of the observations). We then used the training set to estimate the parameters and used the testing set to evaluate the out-of-sample prediction power. To make the model assessment more reliable, we repeated this process 100 times and found that the results are quite robust.

We also used the Area Under the Curve (AUC) metric to evaluate the predictive power of our model. Because the ROC curve is typically close to the upper left corner, the AUC always ranges from 50% to 100%. A larger AUC denotes a better model predictive power. The average AUC for the 100 times of random sampling runs is 78.4%, which is fairly good.

## Discussion and implications

This paper examined the factors that affect driver response behavior to ride-hailing requests, which is highly important for ride-hailing platforms. After controlling for the potential time effects, we found that the economic incentives, the characteristics of the requests *per* *se*, and the spatio-temporal supply-demand intensities are the most influential factors in driver response behavior. The two types of economic incentives are not equivalent: firm subsidies have a much larger effect on driver response behavior than do passenger premiums. This result possibly occurred because the subsidies are randomly offered by the ride-hailing platform, while passenger premiums are determined by the passengers. Consequently, the drivers may infer that a generous premium is offered only when the passenger anticipates difficulty getting a response (i.e., the request is less attractive). To provide evidence for this conjecture, we divided the requests into attractive ones and unattractive ones, and examined the economic incentives associated with these two types of requests. We found that, compared to attractive requests (those that received at least one response), unattractive requests are associated with more generous passenger premiums. Thus, it seems that passengers indeed tend to provide more generous premiums for requests that may be unattractive. Similarly, the drivers may also infer that requests repeatedly submitted to the ride-hailing platform are less attractive; thus, they are less likely to respond to such requests. However, another request characteristic, geographical distance, has a large positive impact on driver response probability.

More importantly, the spatio-temporal supply and demand intensities can also significantly explain driver response behavior. In particular, driver response probability is negatively correlated with the demand intensity but positively correlated with the supply intensity. Statistical tests showed that the magnitude of the demand effect is larger than that of the supply effect on driver response behavior. Finally, the results suggest that the effect of the drivers’ characteristics on their response behaviors is relatively small.

These empirical investigations of ride-hailing services from a driver’s perspective provide new opportunities for ride-hailing platforms to improve their business. First, economic incentives are essential in increasing the driver response rate, especially incentives in the form of firm subsidy. According to our findings, a firm subsidy of 1 RMB effectively increases the driver response probability, and the drivers become less sensitive as the amount of the subsidy increases further. Thus, a policy that fosters a small subsidy for more drivers may be optimal for ride-hailing platforms, allowing companies to effectively increase the driver response rate at a relatively lower cost. In addition, our research demonstrates that passenger premiums also help increase the driver response rate, especially a premium of larger than 5 RMB. Although the amount of the premium is determined by the passengers (rather than the ride-hailing platforms), the platforms can develop algorithms to recommend an appropriate premium amount for the request to obtain a response (considering the nearby demand and supply intensities).

Second, the ride-hailing platforms must consider the dynamic supply and demand intensities at different times and in different locations. Taking advantage of big data and information technology, it is possible for the ride-hailing platforms to reallocate supply on a real-time basis, narrowing the gap between supply and demand over time and across locations. For example, the platforms can learn each driver’s real-time geographical location and past behavior (e.g., when and where the driver usually picks up passengers), and recommend the nearest locations that will have higher ride-hailing demand over the next twenty or thirty minutes. These tactics may not only increase the drivers’ earnings but also enhance passenger loyalty, because passenger requests are more likely to be fulfilled when more drivers are available. Furthermore, the negative impact of excessive demand on driver response behavior is larger than the positive impact of supply. Therefore, the ride-hailing platforms should pay particular attention to locations with excessive demand and allocate drivers from other low-demand areas to these locations in advance.

Third, the ride-hailing platforms may treat the ride-hailing requests differently while developing promotion strategies. Our results show that hailing requests with short geographical distances and those repeatedly submitted by the same passenger are less likely to elicit a response. Thus, the ride-hailing platforms may provide subsidies for these unattractive requests, while providing less or no subsidy for attractive requests (that will elicit responses regardless). In addition, the ride-hailing platforms can also develop a recommendation agent to calculate the response probability of each driver nearby for a particular request and then send the request only to those who are most likely to respond. This may help reduce the number of requests received by a particular driver, which is found to negatively affect their response probability to a subsequent request. Moreover, the ride-hailing platforms may send the less attractive requests to the drivers who have learned to be less picky. In this way, both the driver response rate and the profit of the ride-hailing platforms can be improved.

## Conclusion

Using data provided by an online ride-hailing platform, this paper systematically examines the factors that influence taxi driver response behavior to ride-hailing requests. The results show that the economic incentives offered to the drivers, the characteristics of the ride-hailing requests *per* *se*, and the spatio-temporal supply and demand intensities are all key factors that influence driver response behavior. The findings of this paper can help ride-hailing platforms develop actionable strategies to improve the driver response rate and thus the profit of the platforms. On the other hand, our method is flexible and provides some new thoughts regarding the modeling of similar data from other location-based service providers.

To the best of our knowledge, this research is the first empirical work to examine driver response behavior on ride-hailing platforms. Some limitations remain to be further investigated. First, the data were provided by one ride-hailing service provider and only included the ride-hailing service in Beijing. Using these data, we cannot examine driver response behavior to ride-hailing requests in second- and third-tier cities, which is definitely worthy of future examination. In addition, our data include all the request records on only one working day, which may not allow us to control for other effects such as seasonality and weather. To partially rule out the weather effect, we exploited the weather change on the working day we collected our data. In particular, we run the logistic regressions separately for requests submitted during cloudy hours and sunny hours in the day, and found that the results are quite similar. The detailed results of this robustness check are available upon request from the authors. Second, our research does not consider the competition among different ride-hailing service providers. With the burgeoning of ride-hailing business in China, many different firms may enter the market. The competitive strategies of different ride-hailing service providers may also influence driver response behavior on a particular ride-hailing platform. In order to check if simultaneously using multiple ride-hailing platforms by the drivers would systematically change our conclusions, we kept the 38,072 drivers who have responded to more than 50 requests using the ride-hailing platform that we focus on. These drivers are highly unlikely to use the other ride-hailing platforms at the same time. We re-run the logistic regression and found similar results. These results are also available upon request. Finally, we find that passenger premiums have a relatively small impact, which may be caused by the endogeneity of this variable (i.e., the amount of premium is self-selected by the passengers). We have compared the passenger premium associated with attractive vs. unattractive requests, and found that unattractive requests indeed had much more generous premiums. Future research should incorporate this potentially important issue when examining driver response behavior.

## Supporting information

S1 Table Data used. This is the dataset we used for the logistic model.
(ZIP)
